# The dynamics of food for special medical purposes (FSMPs) utilization in cancer care: from doctor recommendations to online pharmacy procurement

**DOI:** 10.3389/fphar.2024.1393784

**Published:** 2024-07-25

**Authors:** Marius Călin Chereches, Cristian Olimpiu Popa, Hajnal Finta

**Affiliations:** ^1^ Faculty of Pharmacy, “George Emil Palade” University of Medicine, Pharmacy Science and Technology, Târgu Mures, Romania; ^2^ The Institute of International Relations and Area Studies, Faculty of European Studies, Babes-Bolyai University, Cluj-Napoca, Romania

**Keywords:** food for special medical purposes, FSMPs, online pharmacy, cancer nutrition, cancer patients, pharmacy, nutrients

## Abstract

This cross-sectional study conducted in Romania examines the increasing role of online pharmacies in providing Food for Special Medical Purposes (FSMP) to cancer patients. Key findings indicate patient satisfaction with ease of access, significant challenges related to costs, and the critical role of healthcare professionals in guiding FSMP selection. Introduction. As cancer treatments advance, the demand for supportive nutritional care becomes increasingly critical, with FSMPs playing a vital role in patient recovery and quality of life. Methods. Employing a cross-sectional study design, we surveyed cancer patients from Romania to assess their experiences purchasing FSMPs through traditional and online pharmacies. We analyzed the factors influencing their choices, the perceived benefits and challenges of online access, and the role of healthcare professional guidance in their decision-making process. Results. The study reveals that while patients appreciate the ease of access and the broad spectrum of available FSMPs online, they grapple with the financial burden and the need for reliable information to make informed decisions. Through a cross-sectional analysis, we found that the expertise and recommendations of healthcare professionals remain integral to the FSMP selection process, suggesting that digital solutions should enhance, not replace, traditional healthcare interactions. Moreover, our findings highlight a crucial gap in the taste and variety of FSMPs, suggesting that patient adherence could be improved through product innovation. The study found a significant association between the type of medical unit where patients followed their treatment (state-owned clinic) and whether they received recommendations from a specialist oncologist for using FSMPs. Nutritionists’ recommendations were also significantly associated with a higher likelihood of purchasing FSMPs online. Discussions. The study reveals that despite the benefits of digital access, a crucial need exists for regulatory measures and professional oversight to ensure the safe and informed use of FSMPs. The trust placed in healthcare professionals’ recommendations continues to be instrumental in navigating the digital marketplace. Conclusion. This work paves the way for future strategies to integrate online solutions with established healthcare practices to optimize cancer care in the digital age.

## 1 Introduction

The provision of cancer patient care is a dynamic field, and one area where complete treatment regimens are becoming increasingly important is nutrition. In this context, Food for Special Medical Purposes (FSMPs) have become increasingly popular and are an essential supplement to conventional cancer treatments. This paper explores the many variables that lead cancer patients to use FSMPs, highlighting medical experts’ role and the increasing power of internet pharmacies in the decision-making process (Elting et al., 2007; Lalla et al., 2008; Nonzee et al., 2008; Carlotto et al., 2013; Arends et al., 2017).

Conventional cancer treatments, while efficacious, often come with a host of side effects that can severely impact a patient’s nutritional status (Caccialanza et al., 2016). Chemotherapy and radiotherapy can cause nausea, vomiting, taste changes, and reduced appetite. These side effects can lead to malnutrition and a weakened immune system, making the treatment less effective. Therefore, it is important to take decisive action to manage them. (Rondanelli et al., 2020; van de Worp et al., 2020; Liu et al., 2022; Liu et al., 2023). FSMPs are designed to complement the medical treatment of cancer patients by providing specialized nutrition and require special attention concerning which elements to be included (International Special Dietary Foods Industries, 2020; Frydrych et al., 2023).

Food supplements and special food categories register an increased importance everywhere (Travis et al., 2019). The selection and utilization of FSMPs necessitate careful deliberation. The right product must be compositionally appropriate and ensure safe concomitance with existing medications (World Health Organisation, 1991; European Parliament and European Council, 2015). Traditionally, healthcare professionals and local pharmacies have been the bastions of guidance and supply for these specialized products, given the restrictions on their advertisement to the general population (Schindler et al., 2023).

Kristina et al. found that the internet is the primary health and medical information source for Indonesian consumers, significantly influencing consumer behavior and decision-making processes. This trend is especially relevant in FSMPs, where consumers need detailed and reliable information to make informed decisions (Kristina et al., 2019). Another study in Saudi Arabia in 2021 highlighted that a significant percentage of participants (around 60.4%) believed online medicinal products could be safe. However, the study highlighted that the participants perceived the most important risk associated with this practice is the difficulty of distinguishing between registered online pharmacies and other unlicensed commercial websites (Alwhaibi et al., 2021).

The availability and regulatory approval of FSMPs also present a considerable challenge (Ballesteros-Pomar et al., 2022; Folwarski et al., 2022). The process is often stringent, and according to Regulation (EU) 2015/2283 (EFSA Scientific Committee, 2012; Younes et al., 2018; EFSA Panel on Dietetic Products, Nutrition and Allergies, 2021; Turck et al., 2021), novel food is subject to approval. The role of healthcare professionals in guiding cancer patients through the maze of FSMP selection, understanding reimbursement issues, and navigating regulatory waters is thus undeniably vital (Crotty, 2008; Perugini et al., 2022).

With the proliferation of online pharmacies, the avenues for accessing FSMPs have expanded, altering the paradigm of patient care. These digital platforms offer convenience, potentially more comprehensive product ranges, and a degree of anonymity many patients find appealing. Yet, this new mode of access is not without its complexities. While beneficial in terms of ease and availability, online pharmacies introduce several challenges that necessitate thorough examination (Hermosilla et al., 2023).

Online pharmacies have emerged as a pivotal element in managing cancer nutrition. The advantages they offer are manifold. For patients grappling with the taxing nature of cancer treatments, the convenience of online ordering can alleviate the burden of physically visiting a pharmacy. This is particularly beneficial for immunocompromised individuals, for whom exposure to public spaces poses a significant health risk. Additionally, online platforms can offer a broader selection of FSMPs, including products that may not be readily available in local pharmacies (Prashanti et al., 2017; Ahmed et al., 2023). A qualitative study performed in North Carolina (USA) examined the perceived advantages and disadvantages of online grocery shopping among women, infants and children participants, highlighting benefits such as convenience and time-saving and drawbacks like inadequate substitutions and online shopping fees (Pitts et al., 2020).

Digital care platforms enhance oncological care by providing patient information, secure messaging, and patient-reported outcomes. They improve communication, medication adherence, and patient satisfaction despite barriers to their implementation (Hopstaken et al., 2021).

According to a study conducted in Taiwan, the influence of celebrity endorsers and pharmacist professionalism has a positive effect on the perceived attractiveness of dietary supplement brands. The study also indicates a strong correlation between purchase intention and brand trust. Additionally, it was found that the relationship between brand trust and purchase intention is significantly impacted by product knowledge. Overall, these findings suggest that both the professionalism of pharmacists and celebrity endorsements can increase the appeal of dietary supplements, and that a customer’s trust in a brand plays a crucial role in their purchase decision (Chou et al., 2024).

However, the transition to online procurement of FSMPs is accompanied by a spectrum of disadvantages. The lack of direct interaction with healthcare professionals can impede the personalized guidance that is often crucial in choosing the right FSMP. There is also the issue of varying quality control standards across digital platforms, raising concerns about the authenticity and safety of the products purchased. The risk of self-medication without proper consultation may increase, as the ease of obtaining FSMPs online could lead to their misuse or overuse (Fittler et al., 2021; Mackey et al., 2022). Specific guidance regarding identifying credible sources of healthcare information in social media is crucial in the digital age, where misinformation can spread rapidly (Kington et al., 2021).

Despite government regulation, awareness of the risks and rules regarding online medication purchasing (OMP) remains limited, with education and employment being critical factors in OMP knowledge, highlighting the need for educational interventions to ensure safe practices and protect consumers from falsified medications. There is a special need for tailored approaches in digital health communication that consider age and education disparities (Gordon and Crouch, 2019; Nola et al., 2024).

Furthermore, the regulatory oversight of online pharmacies is a subject of ongoing debate. Ensuring compliance with safety standards and preventing the sale of unauthorized products is more challenging in the digital space. This raises questions about the adequacy of current regulations to protect consumers in an ever-expanding online marketplace (Sun et al., 2022).

Integrating online pharmacies into the cancer care model transforms how FSMPs are accessed and utilized. While the benefits of convenience and variety are clear, it is imperative to address the potential downsides. It is essential to strike a balance, ensuring that online pharmacies complement the expertise of healthcare professionals, uphold safety and quality standards, and operate within a robust regulatory framework (Nistal-Nuño, 2022).

Price transparency and comparison are other merits of the online pharmacy model. Patients and caregivers can evaluate options more effectively, making informed choices based on cost and product reviews (Fittler et al., 2022). Moreover, disseminating information online can empower patients with knowledge about the nutritional aspects of their treatment, fostering a sense of control over their health journey (Liu et al., 2020; Al-Taie and Yilmaz, 2021).

This study explores the several aspects that directly affect the recommendation, assessment, and purchase of specialized foods for medical purposes (FSMPs) in the context of internet pharmacies serving cancer patients. By carefully examining these variables, the paper hopes to clarify any possible difficulties or advantages related to the acquisition and application of FSMP products and offer suggestions for enhancing the general standard of care provided to this susceptible patient group.

## 2 Materials and methods

### 2.1 Study design and participants

A cross-sectional study was conducted among cancer patients from Romania between 14 June 2023, to 31 October 2023. The participants were recruited using a convenience sampling method, targeting Facebook users from cancer-related groups to partake in the research. In parallel, cancer patients from the Clinical Department of Medical Oncology of the Mures County Clinical Hospital were also invited to contribute to the study.

The questionnaire was developed based on the literature on FSMPs and online pharmacies, a previous qualitative research project, and advice from cancer care professionals. The 27-item survey collected quantitative data and patient narratives and contained predominantly close-ended and open questions. A pilot test was conducted, and we also used the 1 KA platform tool to test the questionnaire.

### 2.2 Data collection

The instrument utilized for data collection was a structured questionnaire comprising 27 items administered online via the 1 KA platform. Responses were recorded on paper for patients affiliated with the Clinical Department of Medical Oncology of the Mures County Clinical Hospital before being transferred to the digital platform. A cumulative total of 31 questionnaires were collected via this hybrid approach. The protocol for data collection received the necessary approval from the Ethics Committee of the Mures County Clinical Hospital. In total, 148 answers were collected using both routes.

The study examines how people with certain medical conditions, especially cancer, use Foods for Special Medical Purposes (FSMP). It uses a questionnaire-based research method in some hospital settings and through patient-only social media groups.

Using a convenience sampling technique, we recruited participants via a snowball method, including patients and their caregivers. While acknowledging the inherent limitation in achieving a strictly representative sample, we assert that this approach remains methodologically sound given the homogeneity of the target population in terms of medical conditions. Importantly, during data analysis, efforts were made to mitigate potential biases associated with non-random sampling.

### 2.3 Statistical analysis

Descriptive statistics were computed for all survey questions to characterize the sample’s demographics and responses. Furthermore, cross-tabulation analyses, logistic regression and Fisher’s Exact Test were conducted to explore potential associations between variables. The descriptive statistical analysis was performed using the analytics feature of the 1 KA platform (University of Ljubljana, 2024), while other statistical evaluations were carried out using Stata software, version 18 (StataCorp LLC, 2019).

Data preprocessing was performed to handle missing responses coded as −3 (survey dropout), −2 (skipped) and −1 (not answered). These entries were entirely removed from the dataset.

### 2.4 Ethical considerations

The study obtained Institutional Review Board (IRB) approval from both the University of Medicine, Pharmacy, Science and Technology “George Emil Palade” Targu Mures, with the registration number 2367 on 6 June 2023, and the Mures County Clinical Hospital, with the registration number 13238 on 9 August 2023.

## 3 Results

We obtained 148 responses from cancer patients, including online and direct submissions from Mures County Clinical Hospital patients. The questionnaire included questions related to awareness of various brands of FSMPs, recommendation patterns, sources of information, satisfaction, and purchasing of FSMP products. Limited demographics were surveyed to protect the anonymity of cancer patients. The topics followed the insights from qualitative research by authors a few months before, and some results confirm those findings (Chereches et al., 2023a).

About one-third of patients (N = 47) participating in the study said they had used FSMPs in the last 12 months. Patients who answered the open question about the details of their disease mentioned several types of cancer (a total of 15 different kinds of cancer), such as melanoma, breast cancer or stomach cancer, while others mentioned health conditions such as cachexia or post-surgery conditions. The effect of using FSMPs is appreciated as beneficial by the patients (use of FSMPs vs. general health assessment, Fisher’s exact 0.000).

Based on the data presented in Table 1, it can be deduced that a substantial proportion of the study participants underwent chemotherapy, with over 60% (N = 60) of the respondents having received this form of treatment. In addition, many individuals underwent radiotherapy and surgery, while other treatments, such as immunotherapy, were also mentioned frequently.

**TABLE 1:** Q5 What types of treatments did you undergo?

	Frequency	Valid	% - Valid
Chemotherapy	60	96	63
Radiotherapy	36	96	38
Surgery	49	96	51
Other	25	96	26
Total valid		96	

During the survey, we inquired about the type of medical unit in which the participants underwent treatment (Q6). The results revealed that 34% (N = 50) of the participants received treatment in a state-owned hospital, which is a hospital owned and managed by the government. Another 16% (N = 24) of the participants received treatment in a private hospital, which is a hospital that is owned and managed by a private company. Additionally, 11% (N = 17) of participants received treatment in both settings, meaning they received treatment in a state-owned and private hospital.

Table 2 presents the breakdown of the sources that led to the recommendation of using Food for Special Medical Purposes (FSMPs). The data shows that oncology specialists had the highest percentage of recommendations at 39% (N = 23), followed by relatives and acquaintances at 20% (N = 12), pharmacists at 19% (N = 11), and nutritionists and other patients at 17% each (N = 10).

**TABLE 2:** Q10 Who recommended you to use these products?

	Frequency	% Valid
The specialist oncologist	23	39
Relatives and acquaintances	12	20
Pharmacist	11	19
Nutritionist	10	17
Other patients	10	17
General practitioner (GP)	8	14
Someone else	7	12
Radiotherapy specialist doctor	5	8

When asked if they also used other food supplements or vitamins along with FSMPs, 55% (N = 53) of those who answered were positive.

**FIGURE 1:**
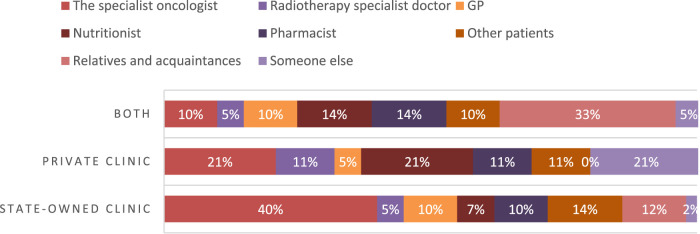
Cross tabulation Q6 Type of the medical unit to get treatment/Q10 Who recommends using FSMPs? Pearson Chi-square (14) = 23.604, p = 0.0511.

Cross-tabulating variables related to the type of medical unit where participants were treated and the source of recommendation show different results, as illustrated in Figure 1. The data shows that patients who received treatment in state-owned medical facilities were more likely to receive recommendations from oncologist specialists. Specifically, 40% of patients treated in these units were given recommendations from oncologist specialists. On the other hand, patients who received treatment in private clinics were more likely to be recommended with FSMPs (Food for Special Medical Purposes) by a wider range of specialists, including nutritionists (21%), radiotherapy doctors (11%), and other types of specialists.

A Fisher’s Exact Test was conducted to examine the relationship between the type of medical unit where patients followed their treatment (Q6) and whether they received recommendations from a specialist oncologist (Q10a). The contingency table for the observed and expected frequencies is presented in Table 3. The Fisher’s Exact Test yielded a p-value of 0.002, indicating a statistically significant association between the type of medical unit where the treatment was followed (state-owned clinic) and whether a specialist oncologist provided recommendations for using FSMPs.

**TABLE 3:** Contingency table for type of medical unit and specialist oncologist recommendations (p = 0.002).

Q6 Type of medical unit	Q10a specialist oncologist (recommendation)		
	No	Yes	Total
1 state-owned clinic	9	17	26
	15.3	10.7	26
2 private clinic	11	4	15
	8.8	6.2	15
3 both	13	2	15
	8.8	6.2	15
Total	33	23	56
	33	23	56

In the context of evaluating patients’ perceptions of using FSMPs, it was found that most patients were generally satisfied with the efficacy, taste, and availability of these products, with ratings of above 3 out of 5 (Question 18 On a scale of 1–5, where 1 is very poor, 3 is fair, and 5 is very good, how do you rate the Food for special medical purposes product that you have used?). However, there is concern regarding the price of these products. The price rating was only 2.7 out of 5, indicating that patients are less satisfied with the cost factor of FSMPs. A summary of the data is presented in Table 4.

**TABLE 4:** Q18 On a scale of 1–5, where 1 is very poor, 3 is fair, and 5 is very good, how do you rate the Food for special medical purposes product you have used?

	1	2	3	4	5	Average	Std. deviation
Efficacy	2	3	24	10	13	3,6	1,06
Taste	4	5	18	16	7	3,3	1,10
Price	9	13	19	4	5	2,7	1,17
Availability	2	9	25	10	3	3,1	0,90

These findings are consistent with the responses to question 24, where 82% of the participants considered FSMPs expensive or extremely expensive products. This indicates that cost is an important patient consideration when using these products. It is important to note that the participants did not express dissatisfaction with the quality or effectiveness of FSMPs, but the cost factor was a significant concern.

As per the research, the respondents were asked to provide their opinions and feedback on the taste and aroma of the FSMPs they used. The researchers asked this question separately to understand their acceptance levels. The question was, “What do you think about the taste and aroma of these products?”. Of all the respondents, 81% responded positively and said they had no significant issues with the taste. They either said, “I had no problems with the taste” or “They are pleasant to the taste”. This means that most respondents were content with the taste and aroma of the FSMPs they used. However, in Question 14 (“Do you think these food products for special medical purposes should be available in more forms and tastes?”), 60% (N = 32) of answers indicated that new tastes and aromas would be helpful. In question 15, we asked participants to share their thoughts on improving FSMP products. We received 25 responses, each suggesting a different improvement, such as enhancing taste and aroma, increasing availability, introducing new formulations like ice cream, offering sugar-free and odourless options, providing powders for shakes, creating hyper-caloric options, and making the products eligible for reimbursement.

According to the study results (Table 5), most individuals seeking information about specialized food for medical purposes (FSMPs) relied on professionals such as doctors and medical staff. This was followed by family members, acquaintances, and other patients. Surprisingly, only 11% of the participants mentioned the internet as a source of information for FSMPs. To further evaluate the accessibility and usefulness of information about FSMPs, the researchers asked question 17. The results showed that 63% of the respondents believed information was available on FSMPs, but it was insufficient.

**TABLE 5:** Q11 How did you learn about Food for special medical purposes products?

	Frequency	% - Valid
The doctor who took care of me recommended them	25	40
The medical staff recommended them to me	20	32
They were recommended to me by relatives or acquaintances	13	21
I learned from other patients	9	15
I found out from the internet	7	11
I found informative materials in the hospital/clinic	5	8
Other sources	5	8

**FIGURE 2:**
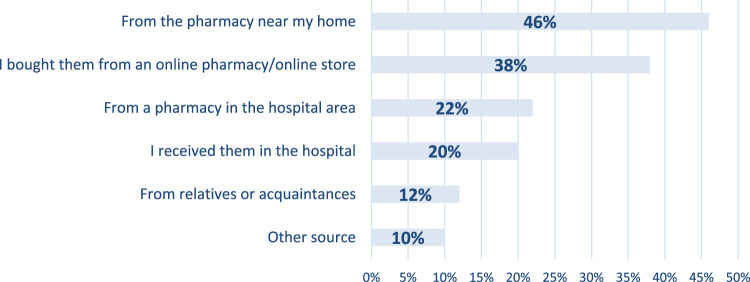
Q19 How did you purchase these products? (n = 50)

The data presented in Figure 2 indicates that almost half of the respondents, 45% of the total, opted to purchase their FSMPs from pharmacies near their homes. On the other hand, 38% of the respondents chose to buy their FSMPs online. Overall, these findings provide valuable insights into the purchasing behaviour of respondents concerning FSMPs.

When asked if they purchased FSMPs online, 54% (N = 29) answered positively (Q20 Did you buy these products from pharmacies/online stores?).

**FIGURE 3:**
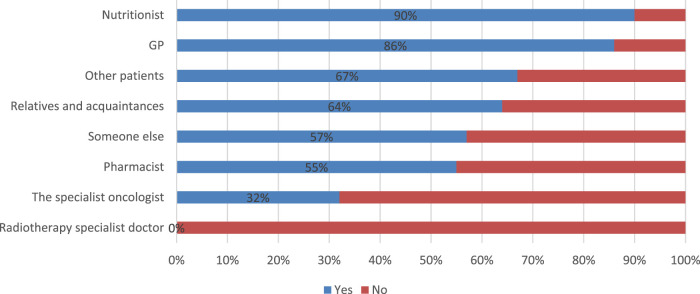
Q20 Did you purchase these products from pharmacies/online stores? Pearson Chi Square(7) = 18.821, p = .00876696.

The results of a cross-tabulation analysis between the question inquiring about the recommendation source for using FSMPs (question 10) and the respondents’ purchase behaviour revealed that those who cited a nutritionist or a GP as their recommendation source were more likely to purchase these products online. Specifically, the analysis showed that 90% of respondents who mentioned a nutritionist and 86% of those who mentioned a GP purchased FSMPs online. These findings are presented in Figure 3.

A logistic regression analysis was conducted to explore the factors influencing the ease of purchasing food for special medical purposes (FSMPs) and the likelihood of buying FSMPs online. The logistic regression analyses provided insights into the impact of recommendations from various sources on these outcomes. The results (Table 6) revealed an odds ratio of 0.1851 for specialist oncologists, suggesting that such recommendations are associated with a lower likelihood of purchasing FSMPs online, with marginal significance (p = 0.086). For GPs, the odds ratio of 5.9903 indicates that recommendations from GPs are associated with a higher likelihood of purchasing FSMPs online, but this is not statistically significant (p = 0.193). With an odds ratio of 12.7149, nutritionist recommendations are significantly associated with a higher likelihood of purchasing FSMPs online (p = 0.044). Recommendations from pharmacists, other patients, relatives and acquaintances, and someone else are not statistically significant. The recommendation of a radiotherapy specialist doctor was omitted from the model, likely due to multicollinearity or lack of variability. This confirms the previous findings.

**TABLE 6:** Logistic Regression Results for Q20 and Q10 (Online Purchase of FSMPs following recommendation).

q20 bin	Odds ratio	Std. err	z	P>|z|	[95% conf. Interval]
Specialist oncologist	0.1851221	0.1817581	−1.72	0.086	.0270224 1.268212
Radiotherapy spec. doctor	1	(omitted)			
GP	5.990257	8.244708	1.30	0.193	.4035446 88.91997
Nutritionist	12.71493	16.02855	2.02	0.044	1.074688,150.4339
Pharmacist	0.9923703	1.04554	−0.01	0.994	.1258537 7.824947
Other patients	1.129039	1.320419	0.10	0.917	.1140835 11.17365
Relatives and acquaintances	1.799807	1.872988	0.56	0.572	.2341069 13.83686
Someone else	0.4852515	0.5693638	−0.62	0.538	.0486654 4.838531
Intercept	1.514801	1.520194	0.41	0.679	.2118963 10.82898

Comparing the answers to questions 22 and 23 reveals a discrepancy that raises concerns about the availability of food for special medical purposes (FSMPs) products in pharmacies. Question 22 asks whether respondents have experienced difficulty procuring FSMPs, to which only 22% of the respondents answered affirmatively. However, question 23 asks respondents to rate the availability of these products in pharmacies, and 56% of the respondents rated it as difficult or extremely difficult. This significant difference in the responses to both questions suggests that other factors may contribute to the perceived difficulty in procuring these products. Further investigation is necessary to determine the discrepancy’s root cause and improve the availability of FSMPs for those who need them.

A logistic regression analysis was conducted to assess the influence of various recommendations on the ease of purchasing food for special medical purposes (FSMPs). The dependent variable Q23 binary was coded for respondents who found it difficult or extremely difficult to purchase FSMPs and 0 for those who found it easy to buy. The logistic regression model did not identify any statistically significant predictors for the ease of purchasing FSMPs. The coefficients for all variables, including recommendations from specialists (oncologists, radiotherapists), general practitioners (GPs), nutritionists, pharmacists, other patients, relatives and acquaintances, and someone else, were not statistically significant (all p-values > 0.05).

According to the responses received, the individuals surveyed shared that they have been frequenting online pharmacies that belong to well-known brands with a solid online presence and a physical retail presence. These brands include Dr.Max, Farmacia Tei, Catena, and Helpnet. Interestingly, it was noticed that a specialized online magazine that caters to special food items, namely, alimentespeciale.ro, was not mentioned at all. On the other hand, emag.ro, a general online shopping platform primarily specializing in electronics, was mentioned alongside online pharmacies, which is quite intriguing. Regional pharmacy chains recognized as low-priced (Ducfarm or Remedium) are also mentioned. For more information, please refer to Table 7.

**TABLE 7:** Q21 Which of the following pharmacies/online stores have you visited in search of food for special medical purposes products? (N = 29).

	Frequency	%
Farmacia Dr.Max (www.drmax.ro)	13	21
Farmacia Tei (comenzi.farmaciatei.ro)	13	21
Catena (www.catena.ro)	10	16
Help Net (www.helpnet.ro)	7	11
eMag (www.emag.ro)	6	10
Other	6	10
Ducfarm (www.ducfarm.ro)	3	5
Spring farma (www.springfarma.com)	2	3
Remedium (www.remediumfarm.ro)	1	2

The assessment conducted on the prices of FSMPs revealed an overwhelmingly negative response from the participants. Among the 41 respondents (82%) perceived the products as being exorbitantly priced or unreasonably expensive. Further, 32 participants (42%) believed that the cost of the products was not justified. However, 85% of valid answers indicated that the monthly cost of FSMPs would be below 500 lei.

Based on the analysis of valid responses, it was found that a considerable proportion of the participants expressed their view in favour of reimbursing FSMPs, either in full or partially.

## 4 Discussions

Overall, the results presented in Table 1 underscore the importance of a multimodal approach to cancer treatment, which considers the patient’s needs and circumstances. This approach can help minimize treatment’s adverse effects while maximizing its efficacy, ultimately improving patient outcomes and quality of life.

The findings of this study are consistent with prior research highlighting the widespread use of chemotherapy in cancer treatment, owing to its effectiveness in targeting rapidly dividing cells. However, it is important to note that chemotherapy is often associated with adverse side effects, which can impact the patient’s quality of life.

Furthermore, the use of radiotherapy and surgery in cancer treatment has also been well-established, with these modalities often used in conjunction with chemotherapy to achieve optimal therapeutic outcomes. Additionally, the emergence of newer treatment options, such as immunotherapy, has shown promise in improving patient outcomes, particularly in cases where other treatments may not be effective.

The information in Table 2 indicates that most of the recommendations were given by healthcare professionals, with the remaining percentage coming from those close to the patient.

Data presented within Figure 1 regarding the cross-tabulation of variables related to the type of medical unit in which participants got treated and the source of recommendation suggests that private clinics provide a more diverse range of medical professionals who are involved in the treatment and care of patients, which may lead to better outcomes for patients. Patients in private clinics are more likely to be presented with FSMPs. These results proved statistically significant (Fisher’s exact 0.004).

These findings highlight the need for further research to explore ways to reduce the cost of FSMPs without compromising their quality and effectiveness. It is important to make these products affordable so patients can benefit from them without financial burden.

This significant association with the Fisher’s Exact Test (Table 3) suggests that the type of medical unit influences the likelihood of receiving recommendations from specialist oncologists. Specifically, patients treated in state-owned clinics are more likely to receive specialist oncologist recommendations than those treated in private clinics or mixed setups.

The choice of FSMPs among cancer patients is significantly influenced by healthcare professional recommendations and medical unit type. Nutritionists’ recommendations are associated with an increasing online purchase intent, and patients treated within state-owned facilities get recommendations from specialist oncologist specialists.

Results regarding sources of information and if these are helpful/enough indicate that although some information may be available, it may not be comprehensive enough to meet the needs of individuals seeking information about FSMPs. Overall, these findings highlight the importance of ensuring that reliable and sufficient information on FSMPs is accessible to those who need it, especially considering that many individuals may not be aware of the existence of such specialized foods and their potential benefits. Pharmacists can help patients with information and services. Proper procedures should be in place to ensure their support is used effectively (Rusu et al., 2022).

Data presented within Figure 2 (source of purchasing FSMPs) suggests a preference for local and convenient options (45%), while 38% purchased online, and this option provides the convenience of online shopping. Still, it may also involve shipping and delivery times.

Based on the data available for cross-tabulation, there appears to be no significant statistical difference between online pharmacies when evaluating prices and availability of products. This means that customers can expect a similar range of products and pricing options regardless of which online pharmacy they choose to purchase from. It is worth noting that this finding is based on comparing multiple online pharmacies, and individual results may vary. However, overall, the data suggests that customers can feel confident in their choice of online pharmacy, knowing that they will likely receive comparable pricing and product availability regardless of which option they select.

The current study offers critical insights into the role of online pharmacies in the distribution of FSMPs to cancer patients, revealing nuanced patterns of utilization and preference. The reliance on well-established pharmacy brands with both online and physical presence underscores patients’ trust in recognized entities within the healthcare system. This trust factor is a cornerstone of healthcare delivery, particularly for cancer patients who require reliable and consistent access to their nutritional aids.

Even though some websites mentioned in a previous study were actively implementing an SEO strategy, they did not receive any mention from the participants who participated in this study. It is worth noting that even though the website was trying to improve its search engine rankings, it did not attract the participants’ attention who were asked to provide feedback on a list of websites (Cherecheș et al., 2023b).

Notably, the assessment of FSMPs’ prices indicates a substantial financial burden on patients, with a vast majority perceiving these products as excessively priced. This perception of high cost is at odds with the necessity for FSMPs in the nutritional management of cancer patients, revealing a gap between the need for and the affordability of these critical products. The study’s findings suggest that despite recognising their utility, FSMPs’ costs could be a barrier to their widespread adoption. This barrier is a significant concern in the broader context of healthcare equity and requires attention from both policymakers and healthcare providers.

Furthermore, our findings regarding the perceived availability and accessibility of FSMPs in pharmacies draw attention to potential disparities in distribution networks. While some patients report ease in procurement, a notable proportion find it challenging, indicating possible geographic and logistical barriers that may prevent consistent access to these essential products. This discrepancy necessitates a more in-depth exploration of distribution practices and a strategy to enhance the availability of FSMPs, ensuring that all patients, irrespective of their location, have equal access to the products they need.

The transition to online pharmacies, while presenting a modern solution to accessibility, carries its own set of challenges. The digital divide, quality control, and the need for regulatory oversight emerge as critical concerns. Moreover, the study’s findings on patients’ satisfaction with FSMPs’ taste and variety suggest that sensory acceptance plays a pivotal role in compliance and long-term utilization, hinting at the need for industry innovation in product development.

The way FSMP products are purchased has a big impact on cancer care. It affects how accessible the products are, the quality of care, how well patients stick to their treatment, and the outcomes of the treatment. Buying online is convenient and can help patients stick to their treatment, while local pharmacies offer important professional support. However, there are challenges in getting FSMP products that can make it hard for cancer patients to get the nutritional support they need.

In light of these observations, future research should delve into the economic impact of FSMPs within the healthcare system, exploring avenues for cost reduction without compromising quality. Additionally, the role of online pharmacies in patient education and decision-making warrants further exploration, particularly in understanding how digital platforms can enhance, rather than hinder, the patient-physician relationship. The potential for misinformation and the challenges of remote guidance are areas ripe for investigation.

Ultimately, this study’s findings contribute to a growing body of literature that seeks to understand the complex interplay between patient care, digital health solutions, and nutritional management in cancer treatment. As the healthcare landscape continues to evolve, so must our approaches to ensuring that patients receive the support they need in the most accessible and sustainable ways possible. The discussions herein serve as a stepping stone for ongoing inquiry and action towards a more equitable and effective healthcare system.

The study has some limitations related to the sample size. Despite being promoted well to patients through online channels, specialized groups, and physically in a hospital, the response rate was low. Therefore, we can only conclude that oncology patients may not be open and willing to share their experiences due to the significant treatment difficulties that are more important to them. Thus, we cannot generalize the results. Another limitation is the restricted sample size (n = 148), which hinders the capacity to apply the findings to a broader population. Subsequent investigations should use more extensive samples to authenticate these findings. The study’s cross-sectional design prevents making causal assumptions. Longitudinal studies are necessary to establish a cause-and-effect relationship between the sources of recommendations and the outcomes. The utilization of self-reported data may introduce bias. Subsequent studies should take into account quantifiable indicators of the availability and purchase of Food for Special Medical Purposes (FSMPs).

Further research is necessary to explore the patterns of utilization of FSMP products and the need for information is evident. Therefore, training and involving healthcare professionals is crucial to make it easier for patients to access these products when their health status demands it.

## 5 Conclusion

This study offers vital insights into the role of online pharmacies in facilitating cancer patients’ access to Food for Special Medical Purposes (FSMPs). It underscores the complex interplay between cost, accessibility, trust, and regulatory considerations that shape patients’ procurement behaviours. The findings reveal a preference for online pharmacies with established brands, indicating trust and perceived quality assurance are critical factors influencing patient choices.

The analysis of FSMP pricing revealed a significant financial burden on patients, with a majority perceiving these products as costly and, at times, prohibitively expensive. This concern is notable given the essential role of FSMPs in supporting the nutritional health of cancer patients. Despite the high costs, most patients reported their monthly expenditure on FSMPs was manageable, suggesting that while FSMPs are considered expensive, they are not out of reach.

In terms of availability, this study highlights a discrepancy in patient experiences, with some reporting challenges in procuring FSMPs from local pharmacies. This indicates a potential gap in distribution and calls for a more consistent supply chain to ensure FSMPs are readily available to those in need, irrespective of their geographical location or financial status.

Notably, the study found that healthcare professionals remain the predominant influencers in recommending FSMPs, reinforcing the need for their continued involvement in guiding patient choices, even in an increasingly digital marketplace. The role of online pharmacies is expanding, but it should complement, not replace, the personalized care and expert advice provided by healthcare providers.

Patients reported satisfaction with the taste and quality of FSMPs, although there is room for improvement in variety and palatability, which may affect long-term adherence to FSMP regimens. The desire for greater variety and the suggestions for product improvements point to opportunities for innovation in the FSMP market.

Healthcare professionals, especially specialized oncologists and general practitioners, significantly influence the ease of obtaining Food for Special Medical Purposes (FSMPs). This suggests a need for improved training and resources to assist patients in obtaining these essential supplies. Policymakers should simplify the distribution of FSMPs and develop strategies to simplify availability. Online platforms linked to nutritionist advice can also enhance the availability of FSMPs, highlighting the need for healthcare personnel to advocate for trustworthy online sources.

While online pharmacies present a convenient and accessible source for FSMPs, there is a clear need for regulatory bodies and healthcare systems to address the issues of cost and equitable access. The digital shift in healthcare provides opportunities for improved patient education and support, but it also challenges that must be managed to ensure patient safety and optimal care. As the healthcare landscape continues to evolve, so must our strategies for integrating digital solutions with traditional healthcare services to support the complex needs of cancer patients. Policymakers should focus on improving labelling and transparency for FSMPs in the online marketplace.

Future research should explore longitudinal patient tracking to understand evolving digital procurement behaviours.

## Data Availability

The raw data supporting the conclusions of this article will be made available by the authors, without undue reservation.
