# Supplementary material for: The dynamics of food for special medical purposes (FSMPs) utilization in cancer care: from doctor recommendations to online pharmacy procurement
Source: Front Pharmacol. 2024 Jul 25;15:1393784. doi: 10.3389/fphar.2024.1393784 (PMC11307204; doi:10.3389/fphar.2024.1393784)
Supplement: Supplementary file 1 [file DataSheet1.PDF]

FSMP for analysis

REPORTS

SURVEY SHORT TITLE:	Alimente pentru uz medical special_ pentru analiza
QUESTION NUMBER:	27
NUMBER OF VARIABLES:	91
STATUS:	Active from: 18.02.2024 Active until: 18.02.2024
AUTHOR:	mariuscalin , 19.02.2024
EDITED:	mariuscalin , 19.02.2024

Q1 - Have you used food products intended for special medical purposes in the last 12 months?

◯ Yes

◯ No

Q2 - Can you give some examples of brands (product names) used?

[ ]

Q3 - Which of the following brands of Food for special medical purposes products are you familiar with?

Multiple answers are possible

	I know it & I used it	I know it, but didn't used it	Neither know nor used
Fresubin	☐	☐	☐
Nutridrink	☐	☐	☐
Medidrink	☐	☐	☐
Nutricomp	☐	☐	☐
Recover Nutrition	☐	☐	☐
Altele	☐	☐	☐

Q4 - What medical condition led you to use these products?

[ ]

Q5 - What types of treatments did you undergo?

Multiple answers are possible

☐ Chemotherapy

☐ Radiotherapie

☐ Surgery

☐ Other: [ ]

Q6 - In which type of medical unit did you undergo the treatment(s)?

◯ state-owned clinic

◯ private clinic

◯ both

Q7 - Have you used the food products for special medical purposes together with other medical treatments or food supplements (vitamins, minerals)?

◯ Yes

◯ No

Q8 - What do you think about the following statements regarding the use of these products?

	Not at all	Partially	Mostly	Most of the times	Full agreement
The products were effective	◯	◯	◯	◯	◯
Health has improved	◯	◯	◯	◯	◯
They helped me to follow and complete the treatment	◯	◯	◯	◯	◯

Q9 - How often have you used these Foods for special medical purposes products?

◯ one per day

◯ two per day

◯ three per day

◯ more than 3 per day

◯ different each other day

Q10 - Who recommended you to use these products?

Multiple answers are possible

☐ The specialist oncologist

☐ Radiotherapy specialist doctor

☐ GP

☐ Nutritionist

☐ Pharmacist

☐ Other patients
☐ Relatives and acquaintances
☐ Someone else ________

Q11 - How did you find out about Food for special medical purposes products?

Multiple answers are possible

☐ The doctor who took care of me recommended them
☐ The medical staff recommended them to me
☐ I found out from the internet
☐ They were recommended to me by relatives or acquaintances
☐ I learned from other patients
☐ I found informative materials in the hospital/clinic
☐ Other sources ________

Q12 - What do you think about the taste and aroma of these products?

◯ The taste is unbearable
◯ The taste is bad, but tolerable
◯ I had no problems with the taste
◯ They are pleasant to the taste

Q13 - What aromas do you consider to be more pleasant / easy to tolerate?

____

Q14 - Do you think these food products for special medical purposes should be available in more forms and tastes?

◯ There are enough aromas and tastes
◯ Other flavors or tastes would be helpful

Q15 - Do you have recommendations for improving food products for special medical purposes products?

____

Q16 - Would you recommend these food products for special medical purposes to other cancer patients?

◯ Yes
◯ No
◯ Depinds on their situation ____________

Q17 - Do you think there is enough information available regarding the use of these foods for special medical purposes?

◯ Yes, there were enough information
◯ There is information, but not enough
◯ No information available

Q18 - On a scale of 1 to 5, where 1 is very poor, 3 is fair, and 5 is very good, how do you rate the Food for special medical purposes product that you have used?

	1	2	3	4	5
Efficacy	◯	◯	◯	◯	◯
Taste	◯	◯	◯	◯	◯
Price	◯	◯	◯	◯	◯
Availability	◯	◯	◯	◯	◯

Q19 - How did you purchase these products?

Multiple answers are possible

☐ I received them in the hospital
☐ From the pharmacy near my home
☐ From a pharmacy in the hospital area
☐ I bought them from an online pharmacy/online store
☐ From relatives or acquaintances
☐ Other source ____________

Q20 - Did you purchase these products from pharmacies/online stores?

◯ Yes
◯ No

IF (1) Q20 = [1]

Q21 - Which of the following pharmacies/online stores have you visited in search of food for special medical purposes products?

Multiple answers are possible

☐ Alimente Speciale (alimentespeciale.ro)

☐ Farmacia Dr.Max (www.drmax.ro)

☐ Catena (www.catena.ro)

☐ Spring farma (www.springfarma.com)

☐ Farmacia Tei (comenzi.farmaciatei.ro)

☐ Help Net (www.helpnet.ro)

☐ Remedium (www.remediumfarm.ro)

☐ Ducfarm (www.ducfarm.ro)

☐ Pilulka (www.pilulka.ro)

☐ eMag (www.emag.ro)

☐ Liki 24 (liki24.ro)

☐ Farmaciile Napofarm (www.farmaciilenapofarm.ro)

☐ Altul: ____________

Q22 - Have you experienced any difficulty in procuring these food for special medical purposes products?

◯ Yes

◯ No

Q23 - How do you rate the availability of these products in pharmacies?

◯ Easy to purchase

◯ Difficult to purchase

◯ Extremely difficult to purchase

Q24 - How do you rate the price of these products?

◯ They are extremely expensive

◯ They are expensive, but we managed

◯ The price is affordable

◯ They are cheap

Q25 - Do you think the price of food for special medical purposes is justified?

◯ Yes
◯ No
◯ Don’t know

Q26 - What would be the monthly cost of food for special medical purposes that you would consider acceptable if you had to pay it out of your own income?

◯ Less than 500 lei
◯ 501-750 lei
◯ 750-1000 lei
◯ Over 1000 lei
◯ Different ____________

Q27 - Do you think these food products should be prescribed and paid (reimbursed) for under the health insurance system?

◯ Yes, in full
◯ Yes, partially
◯ They should not be reimbursed
◯ Don’t know
